# Carboxylated Poly-L-lysine Potentially Reduces Human Sperm DNA Fragmentation after Freeze-Thawing, and Its Function Is Enhanced by Low-Dose Resveratrol

**DOI:** 10.3390/cells12222585

**Published:** 2023-11-07

**Authors:** Ryota Tachibana, Hiroki Takeuchi, Kento Yoshikawa-Terada, Tadashi Maezawa, Mikiko Nishioka, Erina Takayama, Hiroaki Tanaka, Kayo Tanaka, Suong-hyu Hyon, Yuki Gen, Eiji Kondo, Tomoaki Ikeda

**Affiliations:** 1 Department of Obstetrics and Gynecology, Graduate School of Medicine, Mie University, 2-174 Edo-bashi, Tsu 514-8507, Japan; r-tachibana@med.mie-u.ac.jp (R.T.); yoshikawa-k@med.mie-u.ac.jp (K.Y.-T.); tada-m@med.mie-u.ac.jp (T.M.); m-nishioka@med.mie-u.ac.jp (M.N.); eijikon@med.mie-u.ac.jp (E.K.); t-ikeda@med.mie-u.ac.jp (T.I.); 2 Center of Advanced Reproductive Medicine, Mie University Hospital, 2-174 Edobashi, Tsu 514-8507, Japan; erina-t@med.mie-u.ac.jp; 3 Obstetrics and Gynecology, Mie University Hospital, 2-174 Edobashi, Tsu 514-8507, Japan; tanaka-hiroaki@med.mie-u.ac.jp (H.T.); tanaka-ky@med.mie-u.ac.jp (K.T.); 4 BMG, Inc., 45 Minamimatsunoki-cho, Higashikujo, Minami-ku, Kyoto 601-8023, Japan; biogen@bmg-inc.com (S.-h.H.); ygen_bioverde@bmg-inc.com (Y.G.)

**Keywords:** carboxylated poly-L-lysine, cryopreservation, reactive oxygen species, sperm, sperm DNA fragmentation, antioxidant

## Abstract

Sperm DNA fragmentation (SDF) that occurs during the freezing–thawing of sperm may negatively impact the treatment outcomes of assisted reproductive technologies (ART). In a previous study, we developed a human sperm cryopreservation reagent containing carboxylated poly-L-lysine (CPLL) that reduced SDF after freeze-thawing compared with clinically popular cryopreservation reagents containing human serum albumin. However, it is unclear whether CPLL reduces SDF, as it differed from the constituents of the commercial cryopreservation reagents used for comparison. Therefore, here, we examined whether CPLL reduces the SDF of human sperm and evaluated reactive oxygen species (ROS) levels and lipid peroxidation (LPO), which are the causes of SDF; mitochondrial injury, ROS production; and impaired sperm motility. Furthermore, optimal antioxidants and their concentrations that could further enhance the reduction in SDF were determined for future clinical application in ART and underwent the same functional evaluations. CPLL can reduce SDF via inhibition of intracytoplasmic ROS and LPO. Furthermore, the addition of 0.1 mM resveratrol avoided the enhancement of SDF, which potentially affects mitochondrial and cytoplasmic ROS and LPO. This novel human sperm cryopreservation reagent containing CPLL and resveratrol has the potential to improve treatment outcomes in ART using frozen sperm.

## 1. Introduction

Sperm freezing technology, which was developed to preserve animal genetic resources, has found application in the fields of the conservation of endangered and rare species, animal husbandry, and reproductive medicine [1,2,3,4]. To maintain the viability of gametes and reproductive tissues, including sperm, after freezing and thawing, it is important to suppress cell expansion caused by changes in osmotic pressure and the formation of ice crystals inside and outside of cells during freezing [5]. Therefore, the addition of cryopreservation reagents must include cryoprotectants (CPAs), which inhibit osmotic pressure changes and the formation of ice crystals. Currently available human sperm cryopreservation reagents include low-molecular-weight CPAs, high-molecular-weight CPAs, and buffers. The components of human sperm cryopreservation reagents mainly consist of synthetic compounds; however, some of the CPAs used include egg yolk buffer (TYB) [6,7] and human serum albumin (HSA) [8], which are components of animal origin. TYB, compared with other compounds, is rarely used in clinical practice owing to its unstable supply and the risk of zoonotic infections and allergies. HSA-containing sperm cryopreservation reagents are now widely used in clinical practice; however, they are associated with lot-to-lot variation during manufacturing, the risk of allogeneic infections, and unstable supply. To address these problems, we developed the world’s first animal-component-free human sperm cryopreservation reagent using a chemical compound, carboxylated poly-L-lysine (CPLL), as a substitute for animal-derived components [9].

CPLL is an amphoteric electrolyte polymer compound in which a carboxyl group is introduced by reacting a portion of the amino group of ε-poly-L-lysine, which is used as a food additive, with succinic anhydride [10]. Owing to its extremely low cytotoxicity and high cryoprotective properties, CPLL has been used as a cryoprotectant for various cell types, including pig embryos [11], bovine somatic cells [12], and stem cells [13]. Therefore, CPLL could be applied as a substitute for TYB and HSA, which are currently used in human sperm cryopreservation reagents. Our developed human sperm cryopreservation reagent containing CPLL showed comparable performance to HSA-containing sperm cryopreservation reagents upon evaluation of sperm properties, including motility rate and fertilization ability after freezing and thawing; furthermore, it has been suggested that CPLL may reduce sperm DNA fragmentation (SDF), which has recently been the focus of attention [9]. Our novel human sperm cryopreservation reagent had a different composition than the commercially available cryopreservation reagent used as a control, making it unclear whether the reduction in SDF was due to the protective property of the CPLL or not.

SDF is a male infertility factor and a cause of poor embryo quality, such as developmental arrest and fragmentation of embryos by split cells, which has long been a problem in the in vitro fertilization (IVF) of assisted reproductive technologies (ART) [14]. Additionally, SDF may also affect IVF outcomes, such as pregnancy and implantation rates [15,16] and sperm nuclear basic proteins [17,18]. In addition, aging [19], the density gradient centrifugation (DGC) method used in sperm selection [20,21], and sperm freezing have been indicated as causes of SDF [22]. During sperm freezing, SDF is mainly caused by oxidative stress from reactive oxygen species (ROS) produced by mitochondria under osmotic stress [23,24]. In somatic cells, excess ROS is removed by antioxidant enzymes present in the cytoplasm. However, sperms express few antioxidant enzymes, as most are expelled from the cytoplasm just before they are released from the germinal epithelium during formation and are unable to process excess ROS, causing SDF [25]. Furthermore, sperm cell membranes are rich in polyunsaturated fatty acids, which are susceptible to oxidative phosphorylation by ROS [26]; the resulting lipid peroxidation (LPO) in the cell membrane leads to SDF [27]. In humans, sperm plasma containing antioxidant enzymes is cleared by using the DGC method in most cases, and only optimal sperm are frozen. Therefore, it is easy to fall into a state of oxidative stress due to an imbalance between oxidative and antioxidant effects, and the excess ROS generated during freezing cannot be fully processed, producing harmful effects such as SDF and apoptosis [28]. To overcome this problem, many studies have attempted to reduce SDF by adding antioxidant compounds to different sperm cryopreservation reagents [29,30,31,32], although the effects vary depending on the concentration of the antioxidant compound added, the animal species, and the components of the cryopreservation reagents. Furthermore, there have been no reports of synergistic effects with any other antioxidant compounds when added to the human sperm cryopreservation medium containing CPLL.

In this study, we confirmed that SDF was reduced by CPLL and evaluated the contributing factors. Furthermore, to support the clinical application of CPLL for ART, we identified an optimal antioxidant and its concentration to further enhance the SDF-reducing effects of a human cryopreservation reagent containing CPLL and evaluated the associated factors. This study elucidated some of the previously unexplored effects of CPLL on sperm. Furthermore, this is the first report to determine that resveratrol (RES) synergizes with CPLL and enhances the SDF-reducing effects that occur during the freeze-thawing of human sperm.

## 2. Materials and Methods

### 2.1. Ethical Approval

Informed consent was obtained from all participants who donated their semen. Sperm samples used in this study were collected from 24 healthy male partners (average age, 38.0 ± 6.5) of female patients who presented for fertility treatment at our university hospital between April 2020 and April 2022. Only semen that was found to be “healthy” according to the World Health Organization guidelines (2021) was used in the analysis. This study was performed in accordance with the guidelines established by the Clinical Research Ethics Review Committee of Mie University Hospital (H2020-230) and was conducted within the guidelines established by the Ethics Committee of the Japan Society of Obstetrics and Gynecology (No. 104). The study was registered in the University hospital Medical Information Network (UMIN) Clinical Trials Registry in Japan (UMIN000043586).

### 2.2. Cryopreservation Reagents

The human sperm cryopreservation reagent containing CPLL developed in our previous study consisted of modified human tubal fluid (mHTF), 0.3% *w*/*v* CPLL, 7% *v*/*v* glycerol, and 0.1 mM raffinose [9]. To examine the presence or absence of the SDF-reducing effects of CPLL, we used human sperm cryopreservation reagents containing CPLL, without CPLL (CPLL-FREE); we also replaced CPLL with 5% *v*/*v* human serum albumin (HSA; 9301, FUJIFILM, Tokyo, Japan). 

Furthermore, to select an optimal antioxidant compound to reduce SDF, canthaxanthin (CTX) (LKT Laboratories, St. Paul, MN, USA), astaxanthin (AXT) (BLD Pharmatech, Shanghai, China), pyrroloquinoline quinone (PQQ) (KANTO Chemical, Tokyo, Japan), and RES (Tokyo Chemical Industry, Tokyo, Japan) were added to cryopreservation reagent containing CPLL. The final concentration of antioxidants when mixed with sperm ranged from 0.01 pM to 1 mM, as in previous studies [29,31,33,34] (Table 1).

### 2.3. Sperm Preparation, Freezing, and Thawing

In this study, semen was collected via masturbation from healthy men with no male infertility factors. After liquefaction, the semen was immediately examined and subjected to DGC treatment. Only samples that met the conditions of motility > 30% and total sperm count > 100 × 10^6^ cells in accordance with the protocol of our previous study were stored. DGC was performed using 50% and 90% separation media (Irvine Scientific, Santa Ana, CA, USA) at a centrifugal force of 400× *g* for 15 min. The supernatant was discarded, and the sperm was subjected to centrifugation at 400× *g* for 10 min after the addition of a multipurpose processing medium (MHM; Irvine Scientific, Santa Ana, CA, USA) containing 10% *v*/*v* serum substitute supplement (SSS; Irvine Scientific, Santa Ana, CA, USA). After discarding the supernatant, the sperm was resuspended in HTF medium containing 10% *v*/*v* SSS, and the motility rate of the suspended sperm cells was measured. Raw semen and semen subjected to DGC treatment were tested using a computer-assisted sperm analysis (CASA) device, Lens Hooke (Naka Medical Corporation, Tokyo, Japan). Suspended sperm samples that met the conditions of motility >80% and total sperm count >7 × 10^6^ cells were used for subsequent cryopreservation experiments (Table S1). Sperm cryopreservation was performed by adding 250 µL of sperm suspension and an equal volume of cryopreservation reagent to the same cryotube with gentle stirring, resulting in a total volume of 500 µL. The cryovials were allowed to stand for 10 min using a specialized freezing floatation device (Kitasato, Tokyo, Japan) to achieve slow freezing at a height of 3 cm above the liquid nitrogen surface. The frozen sperm samples were stored for approximately 1 week in liquid nitrogen before being thawed for various measurements. The thawing of the cryotubes was performed in a 37 °C water bath until the ice pellet had just melted, which took approximately 2–3 min. The thawed samples were suspended in 5 mL of 10% *v*/*v* SSS/MHM and centrifuged at 400× *g* for 10 min. After discarding the supernatant, the samples were resuspended in 100 µL of 10% *v*/*v* SSS/MHM, and the sperm parameters were measured. Sperm parameters after freezing and thawing were measured using a Makler chamber (Sefi Medical Instruments, Haifa, Israel) because the sperm count was too low to be accurately measured by the CASA. Sperm concentrations and motility rates were calculated using the methods previously described by Bjorndahl et al. [35]. To evaluate at least 200 spermatozoa, all sperm present in 100 grids were counted for each overlapping assessment.

### 2.4. SDF Analysis Using the TUNEL Assay

Freeze-thawed samples were tested for SDF using terminal deoxynucleotidyl transferase dUTP nick-end labeling (TUNEL) assay, as indicated via the APO-DIRECT Kit (BD Biosciences). The TUNEL assay was performed as described by Sharma et al. [36]. Washed samples were adjusted to a density of 1 × 10^6^ sperm and fixed in 1 mL of 4% *w*/*v* PFA at 4 °C for 30 min. The PFA supernatant was centrifuged at 400× *g* for 5 min and discarded. Each pellet was suspended in 1 mL of ice-cold ethanol (70% *v*/*v*) and incubated in the freezer at −20 °C for 30 min. After centrifugation at 400× *g* for 5 min, the supernatant was discarded, and the cells were washed twice with wash buffer. A staining solution was added to the samples, according to the manufacturer’s protocol, and incubated at 37 °C for 60 min in the dark. Raw sperm stained with a staining solution without terminal deoxynucleotidyl transferase was used as a negative control. At the end of the incubation, 1.0 mL of rinse buffer was added to each tube, and each sample was centrifuged at 400× *g* for 5 min. The supernatant was aspirated. Cells were rinsed with 1.0 mL of rinse buffer and centrifuged, and the supernatant was removed by aspiration. The cell pellet was resuspended in 0.3 mL of a propidium/ribonuclease solution. Fluorescence intensity was measured using a FACSCanto II flow cytometer (BD Biosciences, La Jolla, CA, USA). Negative controls were used for all analyses. A minimum of 5000 events per sample were measured using forward and side scatter readings while excluding extra contaminating cells, and a scatter plot was generated by gating only sperm cells. All data, including those on gating the sperm population, were collected and analyzed using FACSDiva software version 6.1.3 (BD Biosciences, La Jolla, CA, USA).

### 2.5. Evaluation of ROS in Mitochondria and Living Sperm

ROS levels were evaluated following the manufacturer’s instructions for commercial kits. For the evaluation of mitochondrial ROS generation in viable cells, MitoSOX Red (MSR; ThermoFisher Scientific, Waltham, MA, USA) and the cell viability stain SYTOX Green (ThermoFisher Scientific, Waltham, MA, USA) were diluted in Hanks’ Balanced Salt Solution (HBSS) (+) and added to cell suspensions containing 1 × 10^6^ sperm to achieve final concentrations of 2 mM and 0.05 mM, respectively. Sperm samples were then incubated at 37 °C for 15 min and subsequently centrifuged at 400× *g* for 5 min. Cell pellets were resuspended in 400 μL of HBSS (+). The MSR and SYTOX Green fluorescence was then measured using a FACSCanto II flow cytometer. Negative controls were used for all analyses. Forward and side scatter readings were set while excluding extra contaminating cells, and a histogram of FITC-A was generated by gating only sperm cells. A histogram of FITC-A was used to measure a minimum of 5000 events of live sperm to generate a histogram of PE-A. Histograms of PE-A were then used to measure MSR-positive sperm. To assess intracellular ROS levels in live spermatozoa, dihydroethidium (DHE; Thermo Fisher Scientific) and the cell viability stain SYTOX Green (Thermo Fisher Scientific) were diluted in HBSS (+) and added to 1 × 10^6^ spermatozoa, resulting in final concentrations of 2 mM and 0.05 mM, respectively. After incubating the cells at 37 °C for 15 min, the samples were centrifuged at 400× *g*. The resulting pellets were resuspended in 400 µL of HBSS (+) and analyzed using flow cytometry as described previously.

### 2.6. Assessment of Lipid Peroxidation

To assess lipid peroxidation, 25 µg/mL anti-4 hydroxy-2-nonenal antibody (4-HNE; NIKKEN SEIL, Fukuroi, Shizuoka, Japan) was added to 1 × 10^6^ sperm suspensions and incubated at 4 °C for 30 min. Spermatozoa were centrifuged at 800× *g* for 5 min and resuspended in Alexa Fluor 647 donkey anti-mouse IgG secondary antibody (ThermoFisher Scientific) diluted 1:500 at 4 °C for 30 min. The samples were washed twice with 1% bovine serum albumin (BSA)/phosphate-buffered saline (PBS). After washing, the samples were resuspended in 300 µL 1% BSA/PBS and analyzed using flow cytometry. Negative controls were used for all analyses. A minimum of 5000 events per sample were measured using forward and side scatter readings while excluding extra contaminating cells. A histogram of APC was generated by gating only sperm cells. Histograms of APC were then used to measure 4-HNE-positive sperm.

### 2.7. Assessment of Mitochondrial Membrane Potential

The mitochondrial membrane potential (MMP) was evaluated using the MitoPT JC-1 assay kit (Immunochemistry Technologies, Davis, CA, USA) following the manufacturer’s protocol. The JC-1 stock solution (Immunochemistry Technologies) was diluted in HBSS (+) and added to 1 × 10^6^ spermatozoa to achieve a final concentration of 2 mM. After a 15 min incubation period, the cells were centrifuged at 500× *g*, and the pellet was resuspended in 300 µL of HBSS (+) for flow cytometry analysis. Immediately prior to analysis, 10 µg/mL PI was added to ensure that only live cells were evaluated. Negative controls were used for all analyses. A minimum of 5000 events per sample were measured using forward and side scatter readings while excluding extra contaminating cells, and a scatter plot was generated by gating only sperm cells. MitoPT JC-1 emits light at 527 nm in normal mitochondria with preserved MMP and at 590 nm with reduced MMP in injured mitochondria. Therefore, scatter plots were generated with FITC on the horizontal axis and PE on the vertical axis, and MMP was measured.

### 2.8. Statistical Analysis

All data were presented as means ± standard deviation. Statistical analyses were performed using GraphPad Prism, v.9 (GraphPad Software). The paired-sample *t*-test was used for two-group comparisons, and one-way ANOVA followed by Tukey’s multiple comparison test was used for three or more group comparisons. Differences with a *p*-value < 0.05 were considered significant.

## 3. Results

### 3.1. Effects of CPLL on SDF

**Figure 1:**
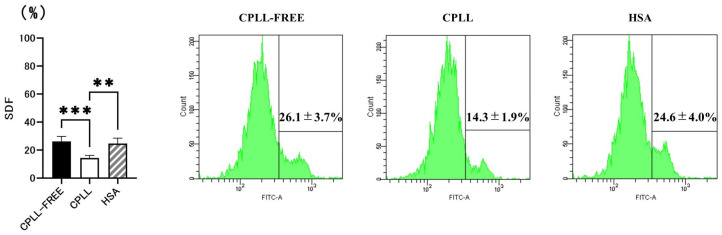
Effects of sperm cryopreservation reagents containing carboxylated poly-L-lysine (CPLL) on sperm DNA fragmentation (SDF) after freezing and thawing of sperm. CPLL-FREE, a human sperm cryopreservation reagent without CPLL. CPLL, human sperm cryopreservation reagent containing CPLL. HSA, human sperm cryopreservation reagent containing human serum albumin. Data are presented as means ± SEM. Statistical significance is indicated by Tukey’s multiple comparison test. Significant differences between groups, ** *p* < 0.01 and *** *p* < 0.001.

Three types of cryopreservation reagents were used to evaluate SDF rates after the freeze-thawing of sperm samples to determine whether CPLLs could reduce SDF: sperm cryopreservation reagent containing CPLL, cryopreservation reagent without CPLL (CPLL-FREE), and cryopreservation reagent with CPLL replaced by 5% *v*/*v* HSA. All sperm specimens used for evaluation had SDF < 30% (Table S1). Sperm cryopreservation reagent containing CPLL significantly reduced SDF compared to other cryopreservation reagents (CPLL-FREE 26.1 ± 3.7% vs. CPLL 14.3 ± 1.9% vs. HSA 24.6 ± 4.0%) (Figure 1).

### 3.2. Effects of CPLL on ROS Levels, LPO, MMP, and Motility Rate

**Figure 2:**
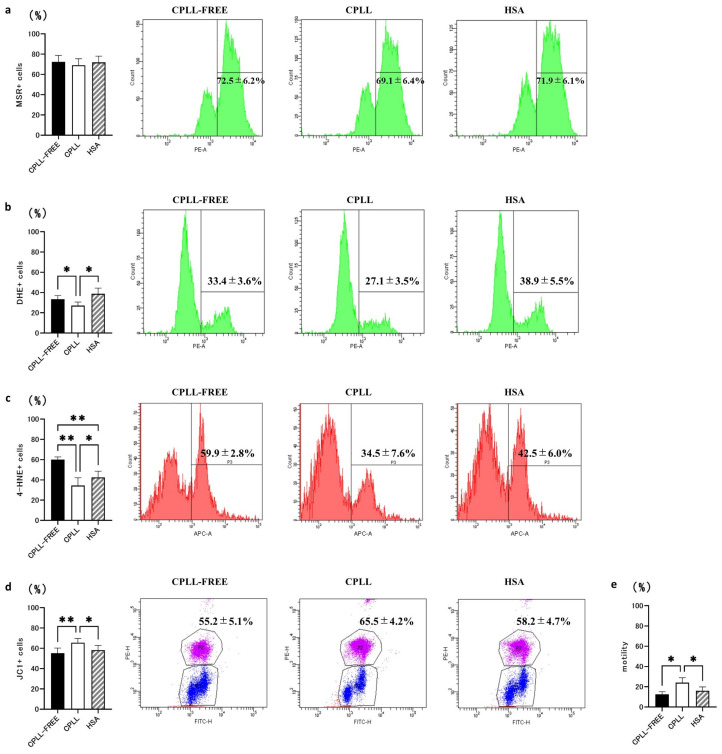
Effects of sperm cryopreservation reagents containing carboxylated poly-L-lysine (CPLL) on reactive oxygen species (ROS) levels, lipid peroxidation (LPO), mitochondrial membrane potential (MMP), and motility rate after freezing and thawing of sperm. (**a**) Mitochondrial ROS level measured by MitoSOX Red (MSR, *n* = 10). (**b**) Total generation of intracellular ROS with dihydroethidium (DHE, *n* = 10). (**c**) LPO state of the sperm membrane measured with the 4-HNE antibody (*n* = 10). (**d**) MMP measured with the JC1 antibody (*n* = 10). (**e**) Sperm motility rate (*n* = 10). CPLL-FREE, a human sperm cryopreservation reagent without CPLL. CPLL, human sperm cryopreservation reagent containing CPLL. HSA, human sperm cryopreservation reagent containing human serum albumin. Data are presented as means ± SEM. Statistical significance indicated by Tukey’s multiple comparison test. Significant differences between groups, * *p* < 0.05 and ** *p* < 0.01.

Next, we evaluated how CPLL affected ROS production and LPO, which are directly implicated in SDF. ROS is produced in mitochondria, and some ROS translocates to the cytoplasm [23]. Therefore, measurements were performed at two locations: the mitochondria and cytoplasm. CPLL did not show a significant difference in mitochondrial ROS (CPLL-FREE 72.5 ± 6.2% vs. CPLL 69.1 ± 6.4% vs. HSA 71.9 ± 6.1%) (Figure 2a), but it significantly reduced cytoplasmic ROS (CPLL-FREE 33.4 ± 3.6% vs. CPLL 27.1 ± 3.5% vs. HSA 38.9 ± 5.5%) (Figure 2b). Furthermore, CPLL significantly reduced the number of 4-HNE positive cells, which is a marker of LPO, oxidative damage to the sperm membrane (CPLL-FREE 59.9 ± 2.8% vs. CPLL 34.5 ± 7.6% vs. HSA 42.5 ± 6.0%) (Figure 2c).

CPLL decreased ROS production; therefore, we evaluated mitochondrial injury, which is a factor that contributes to increased ROS production during sperm freezing. MMP decreases with injury; therefore, MMP was measured as a marker of mitochondrial injury. CPLL achieved significantly higher MMP than the other two groups (CPLL-FREE 55.2 ± 5.1% vs. CPLL 65.5 ± 4.2% vs. HSA 58.2 ± 4.7%) (Figure 2d). As human sperm motility is closely related to ROS and MMP, motility was also evaluated; CPLL showed significantly higher motility, compared to the other two groups (CPLL-FREE 12.5 ± 2.6% vs. CPLL 24.1 ± 4.7% vs. HSA 16.1 ± 3.8%; Figure 2e).

### 3.3. Effects of Different Antioxidant Compounds on SDF

**Figure 3:**
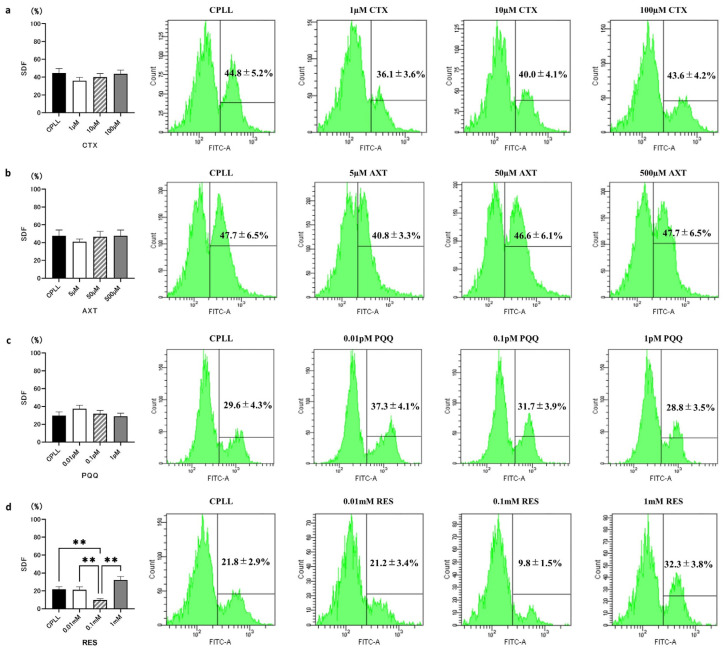
Sperm DNA fragmentation (SDF) rates after freezing and thawing with cryopreservation reagents containing various antioxidant compounds. (**a**) Canthaxanthin (CTX, *n* = 10). (**b**) Astaxanthin (AXT, *n* = 10). (**c**) Pyrroloquinoline quinone (PQQ, *n* = 10). (**d**) Resveratrol (RES, *n* = 10). CPLL, human sperm cryopreservation reagent containing CPLL. Data are presented as means ± SEM. Statistical significance indicated by Tukey’s multiple comparison test. Significant differences between groups, ** *p* < 0.01.

To further reduce SDF, antioxidants were added to the human sperm cryopreservation reagent containing CPLL. We evaluated the effects of various concentrations of the following antioxidant compounds: CTX (1 µM, 10 µM, 100 µM), AXT (5 µM, 50 µM, 500 µM), PQQ (10 nM, 100 nM, 1000 nM), and RES (0.01 mM, 0.1 mM, 1 mM). All sperm specimens used for evaluation had SDF < 30% (Table S1). Sperm cryopreservation reagents with CTX (CPLL 44.8 ± 5.2% vs. 1 µM CTX 36.1 ± 3.6% vs. 10 µM CTX 40.0 ± 4.1% vs. 100 µM CTX 43.6 ± 4.2%) (Figure 3a), AXT (CPLL 47.7 ± 6.5% vs. 5 µM AXT 40.8 ± 3.3% vs. 50 µM ATX 46.6 ± 6.1% vs. 500 µM ATX 47.7 ± 6.5%) (Figure 3b), and PQQ (CPLL 29.6 ± 4.3% vs. 0.01 pM PQQ 37.3 ± 4.1% vs. 0.1 pM ATX 31.7 ± 3.9% vs. 1 pM ATX 28.8 ± 3.5%) (Figure 3c) were not significantly different at any concentration. Sperm cryopreservation reagent with RES showed a significant decrease in the SDF rate with the addition of 0.1 mM RES, compared to the CPLL reagent (CPLL 21.8 ± 2.9% vs. 0.01 mM RES 21.2 ± 3.4% vs. 0.1 mM RES 9.8 ± 1.4% vs. 1 mM RES 32.3 ± 3.8%) (Figure 3d).

### 3.4. Effects of RES on ROS Levels, LPO, MMP, and Motility Rate

**Figure 4:**
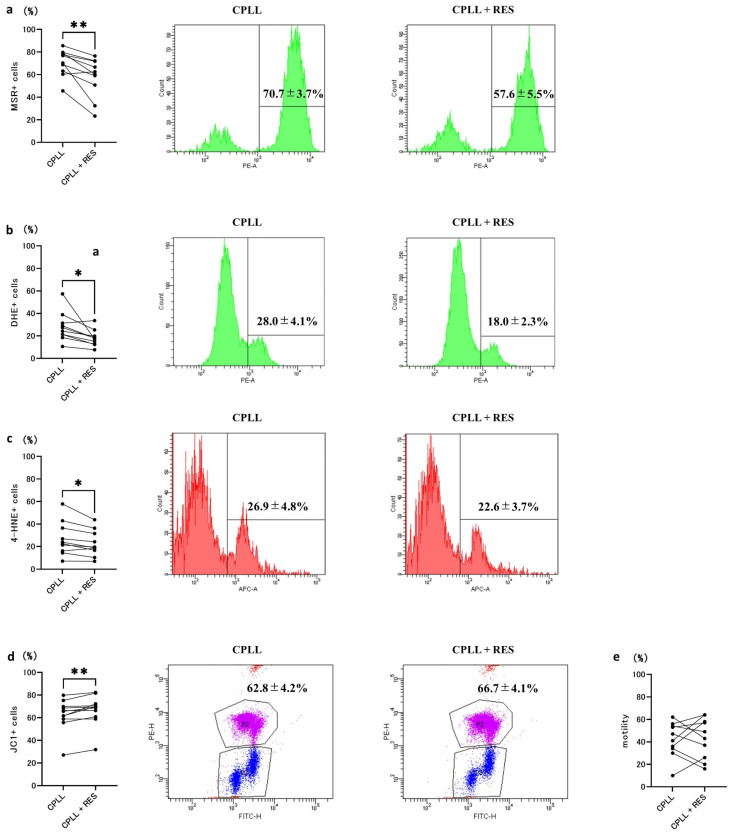
Effects of 0.1 mM resveratrol (RES) on reactive oxygen species (ROS) levels, lipid peroxidation (LPO), mitochondrial membrane potential (MMP), and motility rate after freezing and thawing of the sperm. (**a**) Mitochondrial ROS levels were measured by MitoSOX Red (MSR, *n* = 10). (**b**) Generation of intracellular ROS with dihydroethidium (DHE, *n* = 10). (**c**) LPO state of the sperm membrane measured with the 4-HNE antibody (*n* = 10). (**d**) MMP measured with the JC1 antibody (*n* = 10). (**e**) Sperm motility rate (*n* = 10). CPLL, human sperm cryopreservation reagent containing CPLL. Data are presented as means ± SEM. Statistical significance indicated by Tukey’s multiple comparison test. Significant differences between groups, * *p* < 0.05 and ** *p* < 0.01.

As the addition of 0.1 mM RES decreased the SDF rate, we evaluated ROS and LPO as direct causes of SDF. The addition of RES reduced ROS in mitochondria (70.7 ± 3.7% vs. 57.6 ± 5.5%; Figure 4a) and cytoplasm (28.0 ± 4.1% vs. 18.0 ± 2.3%) (Figure 4b). Furthermore, the addition of RES significantly reduced the number of 4-HNE positive cells, a marker of LPO (26.9 ± 4.7% vs. 22.6 ± 3.6%) (Figure 4c). Furthermore, we evaluated the cause of increased ROS production, which is an indicator of mitochondrial damage. The addition of RES resulted in a significant increase in MMP, which is also a marker of mitochondrial damage (62.8 ± 4.2% vs. 66.7 ± 4.1%) (Figure 4d). The motility rate was also measured to assess the effects of RES; however, no significant differences were observed (42.3 ± 4.9% vs. 43.4 ± 5.7%) (Figure 4e).

## 4. Discussion

Our findings revealed the ability of CPLL to reduce SDF generated during the cryopreservation and thawing of human spermatozoa. Furthermore, the addition of 0.1 mM RES to the human sperm cryopreservation reagent containing CPLL was confirmed to directly or indirectly avoid the enhancement of SDF. These findings suggest that the developed human sperm cryopreservation reagent, when used in clinical applications, may have the potential to reduce SDF in frozen–thawed sperm, thus improving embryonic quality and the outcomes of ART treatments.

The occurrence of SDF during the freezing of human sperm is mainly attributed to rapid osmotic changes during freezing, which are believed to trigger increased ROS production from mitochondria, leading to SDF [23,24]. CPLL used as a CPA in this study has a high affinity for water, suggesting that the polymer solution in which CPLL is dissolved is incorporated into the water remaining in the ice, preventing rapid osmotic pressure changes during freezing [10]. Thus, the use of CPLL as a CPA in human sperm cryopreservation reagents may reduce the generation of SDF during sperm freeze-thawing. Therefore, we compared human sperm cryopreservation reagents with and without CPLL and a human sperm cryopreservation reagent in which CPLL was replaced with HSA. We found that the human sperm cryopreservation reagent containing CPLL reduced SDF and ROS production after freeze-thawing (Figure 1 and Figure 2b). ROS, which are the cause of SDF, are generated during mitochondrial aerobic respiration while producing ATP. If produced in adequate amounts, ROS can easily be managed by the antioxidant enzymes present in sperm and seminal plasma, preventing any adverse effects. However, human sperm often undergo freezing after the removal of seminal plasma, causing excess ROS production during freeze–thaw processes. This excessive ROS production exceeds the limited antioxidant capacity of sperm, damaging mitochondria, the source of ROS, and hindering normal ATP production. Furthermore, inhibition of ATP production causes a leak of unused electrons from mitochondria, leading to increased ROS generation [37]. The increased ROS resulting from this vicious cycle not only leads to SDF but also contributes to LPO in the sperm membrane, which is rich in polyunsaturated fatty acids. LPO is highly detrimental to sperm function, and its elevated levels not only decrease the fluidity of the sperm membrane but also serve as a direct cause of SDF [26,27,38,39]. Therefore, these results suggest that the inhibitory effect of CPLL on osmotic changes may have suppressed mitochondrial injury and reduced ROS production. Furthermore, reduced ROS may result in reduced LPO, leading to reduced SDF generation due to ROS and LPO. The motility rate of the cryopreserved sperm using the human sperm cryopreserved reagent containing CPLL was high after thawing (Figure 2e). Sperm motility is closely associated with LPO and mitochondrial damage. The decrease in cell membrane fluidity and sperm membrane integrity due to increased LPO adversely affects sperm motility [40]. Moreover, sperm motility relies on ATP produced by mitochondria, and mitochondrial damage impairs ATP production, leading to decreased motility rates [38,41]. Therefore, inhibiting LPO and mitochondrial damage is crucial to maintaining high sperm motility rates. CPLL has a low affinity for cell membranes at 37 °C and does not bind to them; however, at lower temperatures, affinity is increased, and it binds to the cell membrane. This reaction occurs rapidly due to temperature changes, resulting in low cytotoxicity, and functions as a protective mechanism against the integrity of the sperm membrane and freeze–thaw damage [42]. Furthermore, CPLL can mitigate rapid osmotic changes, thus exerting a suppressive effect on LPO and mitochondrial damage. It is suggested that these effects may lead to unimpaired sperm membrane fluidity, integrity, and ATP production, leading to high motility rates after freeze-thawing.

Considering the prospective clinical use of ART in the future, we investigated the optimal antioxidants and concentrations to further enhance the reduction in SDF in human cryopreservation reagents containing CPLL. We found that the addition of 0.1 mM RES decreased SDF (Figure 3d). The effectiveness of RES may vary, depending on factors such as animal species, cryopreservation reagent ingredients, and added concentration [43,44]. Many studies have elucidated the mechanism of action of RES as an antioxidant. The free radical scavenging ability of RES is attributable to its phenolic hydroxyl group [45,46]; in addition, its antioxidant effect through the activation of sirtuin 1 (SIRT1) and NF-E2-related factor 2 [43,47] has been reported. Owing to these actions, mitochondrial ROS production is inhibited, subsequently suppressing LPO of the sperm membrane and mitochondrial damage [39]. In our study, the addition of RES also suppressed ROS production (Figure 4a,b), leading to the subsequent inhibition of LPO and mitochondrial damage (Figure 4c,d). The addition of RES improved LPO and MMP, but no change in sperm motility rate was observed (Figure 4e). This is believed to be due to the inhibitory effect of cyclooxygenase (COX) 1 of RES. RES improves mitochondrial function by activating SIRT1, thus increasing the sperm motility rate [30,41,48]. Conversely, RES has a contradictory effect of selectively inhibiting COX1, an enzyme necessary for the synthesis of prostaglandin 2, which is essential for sperm motility. As the concentration of RES increases, it deteriorates sperm motility [49,50]. In this study, although the addition of RES improved LPO and MMP after freeze-thawing, the lack of improvement in sperm motility could potentially be attributed to the COX1 inhibitory effect. However, the lack of a decrease in motility rate may be due to the antagonism between SIRT1 activity and COX1 inhibition, as the RES added concentration was lower than previously reported.

This study had a few limitations. First, there is limited knowledge regarding the effects of CPLL on human cryopreservation reagents. CPLL not only has highly cryoprotective properties but also shows synergistic effects with cryopreservation reagents [51,52]. However, few studies have evaluated the effects of CPLL on human sperm or when used in combination with RES. Here, we demonstrated the ability of CPLL to reduce the SDF generated during the freezing of human sperm. However, the precise mechanism underlying this effect remains elusive. Whether the reduction in SDF stems from the activity of CPLL in preventing osmotic changes, leading to a decrease in ROS, or whether there is a direct removal of ROS has been indirectly inferred but lacks direct evidence. Furthermore, an increase in RES concentration is known to impair sperm motility; however, in this study, the concentration of RES added to the sperm cryopreservation reagent containing CPLL was lower than the standardly used concentration. As a result, sperm motility remained unchanged with the addition of RES to the sperm cryopreservation reagent containing CPLL. Whether the outcome was due to the impact of RES concentration or the synergistic effect of CPLL and RES remains unknown. Further research is essential to elucidate this phenomenon. In addition, it is unclear how the combination of CPLL and RES may affect the cryopreservation of non-sperm somatic cells. Second, the number of compounds examined to enhance the SDF-reducing effect of human sperm cryopreservation reagents containing CPLL is limited. Furthermore, methods with cryoprotective effects, other than adding CPAs to the sperm cryopreservation reagents, have also been described [53,54]. Combining this method with a sperm cryopreservation solution containing CPLL could potentially lead to the development of a more effective sperm cryopreservation solution that may improve sperm parameters, including SDF. Third, we were unable to evaluate the effects of long-term storage in liquid nitrogen. In this study, the cryopreservation of the sperm was evaluated within 1 week. For fertility preservation, sperm may be stored in liquid nitrogen for several years or longer. Therefore, before clinical application, it is necessary to evaluate the impact on sperm due to prolonged storage over an extended period.

## 5. Conclusions

Our study suggests that CPLL may potentially exhibit indirect antioxidant effects by suppressing ROS generation and the LPO of the sperm membrane during the freezing of human sperm. Furthermore, the indirect antioxidant effect of CPLL was higher than that of sperm cryopreservation reagents containing HSA; moreover, the difference between the inhibition of ROS and LPO was associated with the decrease in SDF due to freeze-thawing. We also confirmed that the optimal antioxidant compound for the human sperm cryopreservation reagent containing CPLL, developed in our previous study, was 0.1 mM RES. RES supplements the function of antioxidant enzymes assumed to be deficient in sperm and suppresses excessive ROS production and LPO produced by sperm freezing, which may have directly or indirectly avoided the enhancement of SDF. The human sperm cryopreservation reagent containing CPLL and RES, developed in this study, is expected to be used for clinical applications in the future. Although clinical trials are necessary, there is a possibility that sperm cryopreservation reagents containing CPLL and RES could enhance embryonic quality and improve outcomes in ART treatments.

## Figures and Tables

**Table 1:** Components of cryopreservation reagents used in each experiment, analysis method, and number of samples.

	Reagents	Components	Statistical Analysis
Components of cryopreservation reagents containing CPLL	CPLL	0.3% *w/v* CPLL + 7% *v/v* glycerol + 0.1 mM raffinose	Tukey’s multiple comparing test (*n* = 10)
	CPLL-FREE	7% *v/v* glycerol + 0.1 mM raffinose	
	HSA	5% *v/v* HSA + 7% *v/v* glycerol + 0.1 mM raffinose	
Antioxidant compounds added to cryopreservation reagents	CTX	0.3% *w/v* CPLL + 7% *v/v* glycerol + 0.1 mM raffinose + CTX (1 µM, 10 µM, 100 µM)	
	AXT	0.3% *w/v* CPLL + 7% *v/v* glycerol + 0.1 mM raffinose + AXT (5 µM, 50 µM, 500 µM)	
	PQQ	0.3% *w/v* CPLL + 7% *v/v* glycerol + 0.1 mM raffinose + PQQ (0.01 pM, 0.1 pM, 1 pM)	
	RES	0.3% *w/v* CPLL + 7% *v/v* glycerol + 0.1 mM raffinose + RES (0.01 mM, 0.1 mM, 1 mM)	
Composition of RES and CPLL cryopreservation reagents	CPLL	0.3% *w/v* CPLL + 7% *v/v* glycerol + 0.1 mM raffinose	Paired-sample *t*-test (*n* = 10)
	RES	0.3% *w/v* CPLL + 7% *v/v* glycerol + 0.1 mM raffinose + 0.1 mM RES	

Abbreviations: AXT, astaxanthin; CPLL, Carboxylated poly-L-lysine; CTX, canthaxanthin; HSA, human serum albumin; PQQ, pyrroloquinoline quinone; RES, resveratrol.

## Data Availability

Data available on request due to restrictions, privacy, or ethics. The data presented in this study are available on request from the corresponding author.

## Supplementary Materials

The following supporting information can be downloaded at https://www.mdpi.com/article/10.3390/cells12222585/s1, Table S1: Sperm parameters used in the study.
